# Correlation of Gastroesophageal Reflux Disease Symptoms with Body Mass Index

**DOI:** 10.4103/1319-3767.39618

**Published:** 2008-04

**Authors:** Shamail Zafar, Israr U. Haque, Ghias U. N. Tayyab, Ameed U. Rehman, Adeel U. Rehman, Nusrat U. Chaudhry

**Affiliations:** Department of Gastroenterology and Hepatology, Lahore Medical and Dental College, Lahore, Pakistan; 1 Department of Gastroenterology and Hepatology, Surgimed Hospital, Lahore, Pakistan; 2 Department of Gastroenterology and Hepatology, Ghurki Trust Teaching Hospital, Lahore, Pakistan

**Keywords:** Body mass index, gastroesophageal reflux disease, heartburn, obesity

## Abstract

**Background/Aim:**

To find a correlation between symptoms of gastroesophageal reflux disease (GERD) and body mass index (BMI).

**Materials and Methods:**

A total of 603 patients who presented at Ghurki Trust Teaching Hospital and Surgimed Hospital Lahore with symptoms of GERD, were included and interviewed according to a validated GERD questionnaire. It included questions regarding GERD symptoms and their severity/frequency. Symptoms were defined: “frequent” if occurred daily; “occasional,” if weekly; and “severe,” if they were sufficiently intense to change lifestyle. Height and weight were also recorded and their BMI calculated. We used logistic regression analysis was performed to assess the association between the presence of each specific GI symptom and BMI. The odds ratios (OR) for a given specific symptom and 95% confidence intervals (CI) were computed from the coefficients in logistic regression models.

**Results:**

The prevalence of obesity was 25.3%, while 38.1% were overweight. There was an increase in reporting of GI symptoms in obese individuals compared to those with normal BMI who were taken as reference group. Frequent nausea, vomiting, early satiety, epigastric pain, heart burn, regurgitation, postprandial fullness, and dysphagia were present in 10.4, 5.6, 8.9, 17.2, 10.2, 22.1, 23.5, and 21.7%, respectively, of obese subjects compared to 7.9, 1.2, 6.5, 3.5, 4.4, 11.4, 17.1, and 16.6% of normal BMI subjects. BMI showed a positive relationship with frequent vomiting (*P* = 0.02), epigastric pain (*P* = 0.03), regurgitation of food (*P* = 0.02), heart burn (*P* = 0.002), and postprandial fullness (0.01).

**Conclusion:**

The majority of GERD symptoms have a greater likelihood of occurring with increasing BMI.

Gastroesophageal reflux disease (GERD) is a syndrome with classic symptoms of heartburn and acid regurgitation without any evidence of esophageal mucosal injury at one end and erosive esophagitis and complications of Barrett's esophagus such as esophageal adenocarcinoma at the other end.[1] Gastroesophageal reflux symptoms (GERS) are very common in Asian population, with a prevalence ranging from 50 to 70%.[2] These symptoms occur spontaneously or are precipitated by positioning such as leaning forward, lying down, or physical activity. Food, drink, or drugs that decrease the pressure of the lower esophageal sphincter can also precipitate them. These dietary/life style habits promote reflux through changes in the intragastric, lower esophageal sphincter, and in the intra-abdominal pressures.[1] Besides genetic factors,[3] pregnancy[4] and dietary habits, excessive body weight has been widely recognized as a risk factor for GERS in many population-based studies[1,4,5] but not all.[6,7]

Obesity is a major health hazard worldwide and is becoming a major health threat in Pakistan.[8] There are a number of health hazards associated with obesity, including diabetes, hypertension, cardiovascular disease, arthritis, anesthesia risk, respiratory problems, breast cancer, menstrual abnormalities, ovarian dysfunction along with poor social image and rejection.[9]

Dysregulation of the mechanisms that control food intake and energy expenditure is a key to the development of obesity. There are various hypotheses linking the gut to obesity development. One hypothesis suggests that gastrointestinal (GI) tract is a source of satiation factors, which contribute to meal termination and hence determine meal size. A decreased satiation response to food intake may play a role in the development of obesity.[10] An exception to this hypothesis would be the association between obesity and GERD symptoms, for which a mechanistic role (i.e., hiatal hernia) is assumed to be the cause of the symptoms. An alternative hypothesis is that excess food intake could lead to responses that increase GI symptoms.[11]

Given the potential impact of obesity on GERD, we examined, we examined the prevalence and determinants of GERD symptoms in a sample of volunteers who presented to us with any of the symptoms of GERD. In this report, we present a detailed examination of the potential role of overweightedness and obesity on the frequency and severity of GERD symptoms in our study sample.

## MATERIALS AND METHODS

### Study design and population

In this cross-sectional study, 603 patients presenting with upper GI upper symptoms to the Gastroenterology Clinic at Ghurki Trust Teaching Hospital and Surgimed Hospital, Lahore, were included. Inclusion of study subjects started in September 2004 and it was continued till September 2006.

### Inclusion criteria

Both male and female patients of 16–65 years age group, regardless of prior treatment for reflux-related symptoms, were included in the study group. We used the Gastroesophageal Reflux Questionnaire, which was filled by one of the members of study team during the interview of patients. This questionnaire comprised of 80 questions which were organized into different sections. The first section corresponded to typical GERS, heartburn, and acid regurgitation. We defined heartburn as a burning feeling that rises through the chest and acid regurgitation as liquid coming back into the mouth leaving a bitter or sour taste. Of these 603 subjects, when the answer was in the affirmative, they were then requested to describe the frequency, severity, and duration of the symptoms, as well as their impact on daily activities, work, use of health services, and consumption of medical drugs. Frequency, severity, and time of duration of GERS was defined, in each case, in terms of the individual characteristics of heartburn, acid regurgitation, nausea, vomiting, early satiety, epigastric pain, postprandial fullness, and dysphagia. Symptoms were defined as: “occasional” if they occurred at least weekly; “frequent” if these occurred daily; and “severe” if they were sufficiently intense to change lifestyle.

### Exclusion criteria

Patients with ongoing treatment for peptic ulcer with antisecretory or anti-***Helicobacter pylori*** therapy (proton pump inhibitors, H2 blockers, prokinetics, and antibiotics) were excluded. Concurrent diagnosis of inflammatory bowel disease (IBD), erosive or ulcerative gastric or duodenal lesions diagnosed on endoscopy previously, major psychiatric illness or dementia was also excluded. Pregnant females, those having any metabolic disorder or advanced liver or renal disease patients were also not included.

### Anthropometrical measurements

Body mass index (BMI) has become the measurement of choice for many obesity researchers and health professionals, to measure overweightedness and obesity in adults [Table 1]. The BMI is a direct calculation that describes relative body weight for height, is not gender specific, and is significantly correlated with total body fat content. Anthropometrical measurements were taken using standard apparatuses. A digital scale was used to measure body weight (BW). Subjects were weighed without shoes, in light clothing. Standing body height (BH) was measured without shoes to the nearest 0.5 cm with the use of a commercial stadiometer with the shoulders in relaxed position and arms hanging freely. The BMI was then calculated as BW in kilograms (kg) divided by the square of the BH in meter (m^2^). Individuals with BMI <18.5 were considered to be underweight; between 18.6 to 24.9 as being of normal weight; between 25 to 29.9 as being overweight, while those with BMI of ≥ 30 as being obese [Table 1].

**Table 1:** Categories of BMI to identify health risk

Body mass index	Weight categories	Risk
<18.5	Underweight	Increased risk
18.5–24.9	Normal weight	Least risk
25–29.9	Overweight	Increased risk
30 and over	Obese	
30–34.9	Obese Class I	High risk
35–39.9	Obese Class II	Very high risk
>40	Obese Class III	Extremely high risk

Source: WHO guidelines for body weight classification in adults 2003

### Statistical analysis

We used a logistic regression analysis to assess the association between the presence of each specific GI symptom (the binary dependent variable) and BMI (entered as a continuous independent variable) adjusting for age, gender, alcohol, and tobacco use (past or current smoke history relative to never smoked, family history of GERD, and history of NSAIDs intake). The odds ratios (OR) for a given specific symptom and 95% confidence intervals (CI) were computed from the coefficients in logistic regression models in which BMI was categorized as described above. ***P*** values <0.05 were considered statistically significant. Statistical analyses were performed with SPSS version 11.0 for Windows.

## RESULTS

A total of 603 patients were included, 53.5% (323) were females while males were 46.5% (277). Mean age of the subjects was 37.5 ± 4.9 years. Majority of the subjects were in 35–44 years of age group (203). Fifty percent of them were married and 34% were having family history of such symptoms [Table 2]. The prevalence of obesity (BMI ≥30 kg/m^2^) was 25.3%, while 38.1% were overweight. Table 2 shows the proportion of patients in each BMI category, as a whole and by gender, age groups, education levels, marital status, smoking and alcohol intake, family history of GERD, and NSAIDs intake from the combined questionnaires.

**Table 2:** Body mass index distribution in study population

	Underweight, n = 15	Normal, n = 205	Overweight, n=230	Obese Class I, n = 81	Obese Class II, n = 53	Obese Class III, n = 19
Total (n = 603)	2.4	33.9	38.1	13.4	8.7	3.1
Gender						
Female (n = 323)	1.1	45.9	34.4	15.5	0.9	2.2
Male (n = 277)	0.5	14.5	5.0	19.8	3.2	1.5
Age groups (years)						
25–34 (n = 130)	1.4	45.4	36.6	12.2	1.4	1.4
35–44 (n = 203)	0.4	43.0	35.3	12.0	5.3	2.6
45–54 (n = 200)	0.7	28.8	38.3	24.6	5.6	4.6
55–64 (n = 51)	0.5	28.2	46.1	15.7	5.2	2.0
>65(n=16)	1.8	25.0	46.7	20.7	4.8	1.0
Education						
<High school (n = 374)	0.6	23.6	45.4	20.3	6.0	2.4
High school (n = 118)	1.0	37.9	45.3	11.4	2.3	1.1
>High school (n = 111)	0.6	32.4	38.7	18.9	5.0	3.2
Marital status						
Married (n = 306)	1.0	33.6	41.5	15.3	4.1	2.4
Single (n = 203)	0.9	36.9	39.1	16.0	3.7	1.2
Others (n = 94)	0.7	31.3	44.8	16.7	3.8	1.5
Smoking						
Never (n = 363)	0.0	36.2	33.6	16.5	5.9	5.9
Past (n = 41)	1.3	31.7	34.4	19.4	6.2	3.1
Current (n = 199)	0.8	31.9	41.8	16.9	4.3	2.3
H/O alcohol intake						
Yes (n = 41)	0.0	48.0	50.0	1.8	0.2	0.0
No (n = 562)	2.6	33.7	40.0	15.3	7.7	0.7
Family history of GERD (n = 208)	0.2	25.2	46.4	19.9	5.0	1.9
H/O NSAID intake (n = 102)	0.0	29.4	49.1	15.9	3.7	0.5

There was an increase in reporting of GI symptoms in obese individuals compared to those with normal BMI. Frequent nausea, vomiting, early satiety, epigastric pain, heart burn, regurgitation, postprandial fullness, and dysphagia were present in obese subjects with BMI ≥30 kg/m^2^, compared to those with normal BMI subjects. Furthermore, BMI showed a positive correlation with frequent vomiting (***P*** = 0.02), epigastric pain (***P*** = 0.03), regurgitation of food (***P*** = 0.02), heart burn (***P*** = 0.002) and postprandial fullness (0.01) after adjusting for age, gender, marital status, education level, H/O NSAIDs intake, family history of GERD symptoms, and alcohol and tobacco use. Table 3 shows the prevalence of GI symptoms across BMI categories and the adjusted OR for each symptom using the normal weight group as the reference group. Although we observed increased prevalence of frequent nausea, early satiety, and dysphagia in obese individuals, no significant statistical relationship was found between BMI and these symptoms.

**Table 3:** Prevalence of gastrointestinal symptoms across body mass index categories and adjusted ratio for each symptom using the normal weight group as the reference group

BMI category	Under weight	Normal	Overweight	Obese Class I	Obese Class II	Obese Class III	*P* value
Nausea	7.1	7.9	6.8	10.6	10.7	10.0	NS
OR (95% Cl)	0.7 (0.1-6.2)	Reference	1.3 (0.8-2.3)	1.5 (0.8-2.8)	1.3 (0.5-3.5)	0.7 (0.2-2.8)	
Vomiting	0.0	1.2	1.5	3.0	7.1	6.7	0.02
OR (95% Cl)		Reference	1.8 (0.6-5.5)	2.6 (0.7-8.8)	6.7 (1.7-26.6)	4.4 (0.8-24.9)	
Early satiety	28.6	6.5	6.1	9.8	3.6	13.3	
OR (95% Cl)	5.1 (1.3-20.4)	Reference	1.1 (0.6-2.0)	1.5 (0.8-2.8)	0.4 (0.1-1.8)	1.2 (0.4-4.1)	NS
Epigastric pain	-	3.5	10.3	16.7	15.0	20.0	0.03
OR (95% Cl)	-	Reference	2.1 (0.6-7.3)	3.7 (1.0-13.3)	1.8 (0.3-11.1)	4.3 (0.3-56.9)	
Regurgitation	-	4.4	7.8	5.2	5.6	20.0	0.02
OR (95% Cl)	-	Reference	3.2 (1.0-10.4)	1.1 (0.2-6.2)	1.6 (0.2-15.1)	7.3 (0.6-85.3)	
Heart burn	-	11.4	14.2	25.3	16.0	25.0	0.002
OR(95% Cl)	-	Reference	1.9 (1.0-3.6)	3.9 (1.9-8.0)	1.5 (0.4-5.9)	2.8 (0.5-15.5)	
Dysphagia	-	16.6	17.2	17.0	25.9	22.2	NS
OR(95% Cl)	-	Reference	1.3 (0.7-2.3)	1.2 (0.6-2.5)	1.8 (0.6-5.2)	1.3 (0.2-6.8)	

Values expressed in percentages. OR = Odd's ratio, *P*-value <0.05 is taken significant, BMI - Body mass index

## DISCUSSION

We found a strong positive association between obesity and frequent GERD symptoms. These associations remained robust even after adjustments for several important potential confounding factors including age, sex, smoking and alcohol intake, marital status, educational level, and NSAIDs intake. There was an increase in reporting of GI symptoms in obese individuals compared to those with normal BMI. Frequent nausea, vomiting, early satiety, epigastric pain, heart burn, regurgitation, postprandial fullness, and dysphagia were present in obese subjects with BMI ≥ 30 kg/m^2^, compared to those with normal BMI subjects. However, BMI showed a positive correlation with frequent vomiting, epigastric pain, regurgitation of food, heartburn, and postprandial fullness only. Taken together, these findings strongly indicate that overweight and obesity are significant risk factors for GERD symptoms.

Our findings augment a growing body of literature addressing the association between BMI and GERD.[12–15] A recent meta-analysis demonstrated a dose-response relationship between BMI and the risk of reporting symptoms of GERD among both men and women.[16] However, the reference groups in these studies included participants with a BMI of <24 and, therefore, were unable to define the risk of symptoms of GERD among normal weight persons. It should be noted that there are three large-scale studies which have found no significant relationship between BMI and symptoms of GERD.[17–19] Among these a large telephone survey limited to people with symptoms of GERD revealed a dose-response relationship between quartiles of BMI and a daily frequency of symptoms; however, this study lacked asymptomatic controls. A Swedish population-based study revealed that there was no association between BMI at age 20, BMI 20 years before the interview or maximum adult BMI and occurrence of reflux symptoms and thus concluded that GERS occur independently of BMI and weight reduction may not be justifiable as an antireflux therapy.[19]

Our study observed a greater percentage of obese individuals reporting nausea than in normal BMI individuals, but no significant statistical association was observed between BMI and the presence of frequent nausea. The absence of this statistical relationship suggests increased body mass may not be an independent risk factor for nausea and other factors may account for the observed increase in nausea in obese individuals. The absence of a significant relationship suggests that increased body mass may not be an independent risk factor for nausea, and that other factors may account for the observed increase in nausea in obese individuals. Similar observations were made in relation to symptoms of early satiety and dysphagia. However, epigastric pain, heartburn, regurgitation of food, and postprandial fullness were significantly more common in overweight and obese individuals.

Contrary to previous observations of decreased perception of satiation in obese people,[20] we did not observe a significant reduction in the prevalence of reported early satiety with increased BMI. Nevertheless, our data are still consistent with a potential 50% reduction in the odds of early satiety in overweight and obese people compared to normal weight individuals, as expressed by the 95% CI of the estimated OR for early satiety in overweight and obese participants. Interestingly, we found that the maximum report of early satiety was from underweight group of patients.

Crowell ***et al.*** observed more frequent upper and lower GI symptoms in overweight females attending a weight management center compared to normal weight women recruited from the community.[21] Aros[14] did try to find an association between reported symptoms of GERD and BMI in US population and found almost similar values as of the present study in obese persons.

Work done by Jacobson ***et al***.[22] on a large cohort of female subjects also demonstrated the positive relation between reported GI symptoms and BMI and further proved that weight gain was associated with an increased risk of symptoms of GERD and weight loss was associated with a decrease in risk.

While our study adequately demonstrates the relationship between the increase in symptoms of GERD with increasing BMI, it fails to shed any new light on the possible mechanism involved. Previous studies have identified a number of mechanical and hormonal factors. Mechanical factors include increased intragastric pressure, increased transient lower esophageal relaxations, and formation of hiatus hernia.[12,16] Similarly, hormonal factors like leptin, insulin, growth factors, and estrogen have also been shown to contribute to obesity and ultimately to GERD.

In summary, our work suggests that an increasing BMI correlates with a greater risk of having GERD symptoms. Emphasis must be laid on physician advice to lose weight along with other lifestyle modifications and the use of medication. Our findings have important implications for future studies since even moderate weight gain may cause or exacerbate symptoms of GERD.
